# 5-(Carbamoylmethylene)-oxazolidin-2-ones as a Promising Class of Heterocycles Inducing Apoptosis Triggered by Increased ROS Levels and Mitochondrial Dysfunction in Breast and Cervical Cancer

**DOI:** 10.3390/biomedicines8020035

**Published:** 2020-02-18

**Authors:** Biagio Armentano, Rosita Curcio, Matteo Brindisi, Raffaella Mancuso, Vittoria Rago, Ida Ziccarelli, Luca Frattaruolo, Marco Fiorillo, Vincenza Dolce, Bartolo Gabriele, Anna Rita Cappello

**Affiliations:** 1 Department of Pharmacy, Health and Nutritional Sciences, University of Calabria, Via P. Bucci, 87036 Rende (CS), Italy; biagio30@live.it (B.A.); rosita.curcio@unical.it (R.C.); matteo_brindisi@libero.it (M.B.); vittoria.rago@unical.it (V.R.); vincenza.dolce@unical.it (V.D.); 2 Laboratory of Industrial and Synthetic Organic Chemistry (LISOC), Department of Chemistry and Chemical Technologies, University of Calabria, Via Pietro Bucci 12/C, 87036 Arcavacata di Rende (CS), Italy; raffaella.mancuso@unical.it (R.M.); idaziccarelli@gmail.com (I.Z.); bartolo.gabriele@unical.it (B.G.); 3 Translational Medicine, School of Environment and Life Sciences, Biomedical Research Centre (BRC), University of Salford, Greater Manchester M5 4WT, UK

**Keywords:** oxazolidinones, apoptosis, mitochondria, ROS, anticancer compounds

## Abstract

Oxazolidinones are antibiotics that inhibit protein synthesis by binding the 50S ribosomal subunit. Recently, numerous worldwide researches focused on their properties and possible involvement in cancer therapy have been conducted. Here, we evaluated in vitro the antiproliferative activity of some 5-(carbamoylmethylene)-oxazolidin-2-ones on MCF-7 and HeLa cells. The tested compounds displayed a wide range of cytotoxicity on these cancer cell lines, measured by MTT assay, exhibiting no cytotoxicity on non-tumorigenic MCF-10A cells. Among the nine tested derivatives, four displayed a good anticancer potential. Remarkably, **OI** compound showed IC_50_ values of 17.66 and 31.10 µM for MCF-7 and HeLa cancer cells, respectively. Furthermore, we assessed **OI** effect on the cell cycle by FACS analysis, highlighting a G1 phase arrest after 72 h, supported by a low expression level of Cyclin D1 protein. Moreover, mitochondrial membrane potential was reduced after **OI** treatment driven by high levels of ROS. These findings demonstrate that **OI** treatment can inhibit MCF-7 and HeLa cell proliferation and induce apoptosis by caspase-9 activation and cytochrome c release in the cytosol. Hence, 5-(carbamoylmethylene)-oxazolidin-2-ones have a promising anticancer activity, in particular, **OI** derivative could represent a good candidate for in vivo further studies and potential clinical use.

## 1. Introduction

Cancer is one of the main causes of morbidity and mortality in the world. In 2015, it was responsible for 8.8 million deaths, and the number of new cases is predicted to increase by about 70% over the next 20 years [1]. Several of the most widespread cancer types, such as cervical, breast, oral, and colorectal tumors have high cure rates when early diagnosed and treated. Surgery and radiation represent useful treatments, but their use is limited by the ability of many solid tumors to spread to different parts of the body. Furthermore, metastatic process frequently occurs before the primary cancer is diagnosed [2].

Consequently, the research aimed at discovering new useful chemotherapeutic agents is still very important. In this regard, various Food and Drug Administration (FDA)-approved antibiotics, used to treat bacterial infections, have arisen as new promising anticancer drugs; in particular, some of them were proved to target mitochondria in Cancer Stem-like Cells (CSCs), selectively inducing cancer cell death [3,4,5]. This event could be understood considering that mitochondria originally evolved from endosymbiotic bacteria, so it is likely that an inhibition of the mitochondrial function could trigger cell death [3].

On this basis, oxazolidinones might represent an interesting class of synthetic compounds useful in cancer treatment. In fact, they are known to be potent antibacterial agents, used against multidrug-resistant gram-positive bacteria [4]. These synthetic compounds bind to the ribosomal 50S subunit by inhibiting the initiation phase of bacterial translation [5]. Linezolid was the first member of this class to be approved in 2000 by FDA and clinically used for the treatment of gram-positive infections [6], especially those caused by Methicillin-resistant Staphylococcus aureus and Vancomycin-resistant Enterococcus faecalis [7]. Since Linezolid-resistant bacteria began to appear, several structural modifications were performed in order to synthesize and test new molecules [7]. Resistance cases were associated with 23S rRNA mutations during treatment [8] or with the presence of the resistance genes cfr or optrA [9].

During the last decades, many studies highlighted the ability of oxazolidinones to exert multifaceted biological activities, since they are able to interfere with factor Xa [10] aldose reductase [11], HIV [12], metabotropic [13], and glutamate receptor [14]. Furthermore, some derivatives exhibited antidiabetic [15,16], anticonvulsant [17], and anticancer activity [18,19,20,21]. Moreover, further literature data indicate that different oxazolidinone derivatives are endowed with promising antineoplastic activity, in particular by acting as partial agonists of peroxisome proliferator-activated receptor-gamma (PPAR-γ) [22]. Other oxazolidinones, derived from linezolid 3, inhibit cell proliferation inducing apoptosis by caspase-3 and -9 activation in DU145 prostate cancer cells [18], while different 2-thioxo-oxazolidin-4-one derivatives exhibited anticancer activity on hematopoietic cancer cells, and significantly increased the expression of genes involved in inflammation and apoptosis in acute leukemia cells [23]. Very recently, dehydroabietic acid oxazolidinone hybrids have shown to exert a promising antiproliferative activity on several human cancer cell lines, and a particular compound is able to induce apoptosis by causing an arrest of the cell cycle in the G1 phase [24]. Thus, development and evaluation of new oxazolidinones as possible anticancer agents is a very interesting ongoing research topic [23,25,26].

**Scheme 1:**
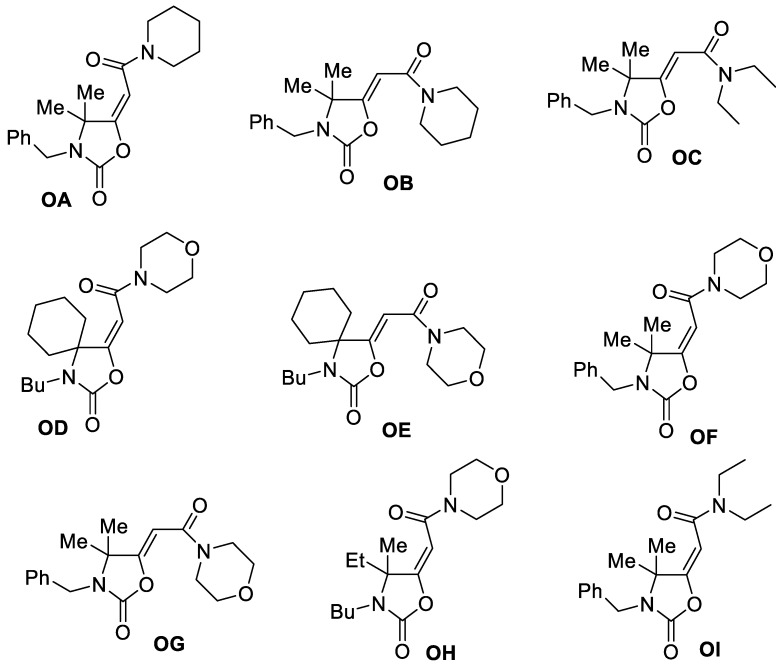
The in vitro anticancer properties of 5-(carbamoylmethylene)-oxazolidin-2-ones (OA-OI).

In the present work, we evaluated the in vitro anticancer properties of some particular oxazolidinone derivatives, which are 5-(carbamoylmethylene)-oxazolidin-2-ones (**OA-OI**) shown in Scheme 1, against estrogen receptor-positive (ER+) breast cancer (MCF-7) and uterin cervix adenocarcinoma (HeLa) cell lines. These compounds were recently synthesized in our laboratories by water-promoted, palladium-catalyzed oxidative carbonylation of α,α-dialkyl-substituted propargylamines [27,28], and have not hitherto been tested for their anti-proliferative potential. We have found that some of these derivatives possess a promising anti-proliferative activity, and that a particular compound ((E)-2-(3-benzyl-4,4-dimethyl-2-oxooxazolidin-5-ylidene)-*N*,*N*-diethylacetamide, (**OI**) is able to induce apoptosis through the mitochondrial intrinsic pathway, increasing ROS production and affecting mitochondrial functions.

## 2. Experimental Section

### 2.1. Synthesis of oxazolidinones OA-OI

5-(Carbamoylmethylene)-oxazolidin-2-ones OA-OI were synthesized by water-promoted, palladium-catalyzed oxidative carbonylation of α,α-dialkyl-substituted propargylamines carried out in the presence of dialkylamines as external nucleophiles, as we already reported [27,28]. Briefly, a 35 mL stainless steel autoclave was charged with PdI_2_ (8.3 mg, 0.023 mmol), KI (38.3 mg, 0.23 mmol), the propargylic amine derivative (1.15 mmol), and the dialkylamine (5.75 mmol) dissolved in the ionic liquid 1-ethyl-3-methylimidazolium ethyl sulfate (EmimEtSO_4_) (2.3 mL). Water (103.5 µL, 5.75 mmol) was then added, and the autoclave was sealed and pressurized at 20 atm (16 atm CO and 4 atm air). After stirring at 100 °C for 24 h, the autoclave was cooled and degassed. The mixture was then extracted with Et_2_O (6 × 4 mL), the collected ethereal phases were concentrated under vacuo, and the products purified by column chromatography on neutral alumina using hexane:AcOEt from 95:5 to 6:4 as the eluent.

### 2.2. Cell Cultures

Human uterin cervix adenocarcinoma (HeLa), human breast cancer ER+ (MCF-7), and MCF-10A human mammary epithelial cells were all purchased from American Type Culture Collection (ATCC, Manassas, VA, USA).

HeLa and MCF-7 cells were maintained in DMEM-F12 medium (Sigma-Aldrich, Milan, Italy) containing 10% fetal bovine serum (FBS, Sigma-Aldrich, Milan, Italy), 2 mM L-glutamine (Sigma-Aldrich, Milan, Italy) and 1% penicillin/streptomycin (Sigma-Aldrich, Milan, Italy), as previously reported [29]. MCF-10A cells were cultured in DMEM-F12 supplemented with 10% horse serum (HS, Sigma-Aldrich, Milan, Italy) [30], 2 mM L-glutamine, 1% penicillin/streptomycin, 0.5 mg/mL hydrocortisone (Sigma-Aldrich, Milan, Italy), 20 ng/mL human epidermal growth factor (hEGF, Sigma-Aldrich, Milan, Italy), and 0.1 mg/mL cholera enterotoxin (Sigma-Aldrich, Milan, Italy). All the tested cell lines were grown at 37 °C in 5% CO_2_ in a humidified atmosphere and periodically screened for possible contamination. All the tested compounds were dissolved in dimethylsulfoxide (DMSO, Sigma-Aldrich, Milan, Italy), and opportunely diluted in DMEM/F12 medium supplemented with 2% FBS in order to achieve working concentrations.

### 2.3. Cell Viability Assay

Cell viability was assessed by using 3-(4,5-dimethylthiazol-2-y1)-2,5-diphenyltetrazolium bromide (MTT) assay (Sigma-Aldrich, Milan, Italy), as already reported [31]. In brief, MCF-7 and HeLa cells (2 × 10^4^ cells/well) were seeded in a 48-well plate and grown in complete medium overnight, then they were switched into DMEM-F12 supplemented with 2% FBS and treated with increasing concentrations (1, 10, 50, 75, and 100 µM) of different oxazolidin-2-ones derivatives (**OA-OI**) or doxorubicin for 72 h. Untreated cells were added with the vehicle (DMSO) and used as a control. After treatment, fresh MTT reagent (used at a final concentration of 0.5 mg/mL) was added, prolonging incubation for 2 h according to the manufacturer’s protocol. Absorbance at 570 nm was measured in each well, including controls, and every experiment was carried out in triplicate.

For each sample, mean absorbance was expressed as a percentage of the control and plotted vs. drug concentration, in order to determine the IC50 value (i.e., drug concentration able to decrease cell viability by 50% with respect to the control) for each cell line [32], generating a sigmoidal dose–response curve by the use of GraphPad Prism 7 software (GraphPad Inc., San Diego, CA, USA). Mean values and standard deviations (SD) of three independent experiments carried out in triplicate were reported.

In order to examine the cytotoxic effects of the compound **OI**, cell proliferation assay was assessed in the presence of N-acetylcysteine (NAC, Sigma-Aldrich, Milan, Italy), a reactive oxygen species (ROS) scavenger, as described in [33]. Briefly, MCF-7 and HeLa cells treated with 15 and 30 µM **OI**, respectively, were incubated with or without 3 mM NAC. After 72 h, cell viability was evaluated by MTT assay.

### 2.4. Cell Cycle Analysis

In order to assess cell cycle analysis, MCF-7 and HeLa cells were seeded in 6-well plates, at a density of 2.5 × 10^5^ cells/well, then they were added to DMSO or treated using 15 and 30 µM **OI**, respectively, for 24 h. MCF-10A were seeded as described before, then they were added to DMSO or treated using 30 µM **OI**, the highest concentration used for cancer cells, for 24 h. Next, cells were harvested by trypsinization, rinsed twice with cold PBS, and pelleted by centrifugation at 1500 rpm for 5 min. Pellets were re-suspended in 70% ice-cold ethanol for 30 min at 4 °C to perform cell fixation. Then, samples were rinsed twice with PBS and stained using a solution containing 3.8 mM sodium citrate (Sigma-Aldrich, Milan, Italy), 100 μg/mL RNAse (Sigma-Aldrich, Milan, Italy), 50 μg/mL propidium iodide (PI, Sigma-Aldrich, Milan, Italy), and 0.1% Igepal (Sigma-Aldrich, Milan, Italy) in PBS for 1 h at 37 °C. Subsequent cytofluorimetric analysis [34] was achieved by BD FACSJazz™ Cell Sorter (Becton Dickinson, Franklin Lakes, NJ, USA).

### 2.5. Mitochondria Extraction

Both MCF-7 and HeLa cells were cultured in 100 mm dishes (1 × 10^7^ cells/dish), added with the vehicle alone (DMSO) or treated for 24 h with 15 and 30 µM **OI**, respectively. Then, mitochondria extraction was performed as previously described [35]. In brief, cells were harvested, resuspended in isolation buffer for cells (IBC, containing 200 mM sucrose, 10 mM Tris/MOPS, 1 mM EDTA/Tris, pH 7.4), and homogenized by a glass potter homogenizer. Nuclei and unbroken cells were removed by centrifugation (600× *g* for 10 min). Supernatants were centrifuged for 30 min at 10,000× *g*, obtaining upper cytosolic fractions and mitochondrial pellets; the latter were resuspended in RIPA buffer (PBS pH 7.4, 1% NP-40, 0.5% sodium deoxycholate, and 0.1% sodium dodecyl sulfate) containing a protease and phosphatase inhibitor cocktail (Sigma-Aldrich, Milan, Italy). Protein concentration was determined using Bio-Rad Protein Assay (Bio-Rad Laboratories, Hercules, CA, USA) and Western blotting was performed as next described.

### 2.6. Western Blot Analysis

Western blot analysis was conducted basically as previously reported [36]. In detail, MCF-7 and HeLa cells were grown to 70–80% confluence, added to DMSO, or treated with 15 and 30 µM **OI**, respectively, for 24 h. In order to obtain total cell lysates, harvested cells were lysed using 200 μL of lysis buffer as previously reported. Cytosolic and mitochondrial fractions were isolated as already described. Identical amounts of proteins from total lysate, mitochondrial, or cytosolic fraction were resolved on SDS-polyacrylamide gel, blotted to nitrocellulose membrane, and probed with proper primary antibodies (Santa Cruz Biotechnology, Dallas, TX, USA) [37]. Immunoreactive products were identified by using ECL Western blotting detection system (Bio-Rad Laboratories, Hercules, CA, USA) [38].

### 2.7. Mitochondrial Staining

Mitochondrial activity was assessed using MitoTracker Orange (Invitrogen, Milan, Italy), a fluorescent dye that enters mitochondria in a membrane potential-dependent way. Mitochondrial mass was determined using MitoTracker Deep-Red (Invitrogen, Milan, Italy), a far red-fluorescent dye that is largely used to label mitochondria. Both MCF-7 and HeLa cells were treated using 15 and 30 µM **OI**, respectively, for 24 h. Control cells were added with DMSO and simultaneously processed [39]. Then, cells were incubated in the dark for 30–60 min at 37 °C with pre-warmed MitoTracker staining solution (diluted in PBS/CM to reach a final concentration of 10 nM). All the next steps were still carried out in the dark. Cells were rinsed in PBS, harvested, and re-suspended in 300 μL of PBS/CM. Cells were then examined by flow cytometry using Fortessa (BD Bioscience, San Jose, CA, USA). Data analysis was performed using FlowJo software (Tree Star Inc., Ashland, OR, USA).

### 2.8. ROS Production

Intracellular level of reactive oxygen species (ROS) was assessed by using CM-H_2_DCFDA (Invitrogen, Milan, Italy), a cell-permeable fluorogenic probe, which is deacetylated by intracellular esterases and can be oxidized in the presence of intracellular ROS, becoming highly fluorescent [35]. MCF-7 and HeLa cells were seeded into 96-well tissue culture plates at a density of 5 × 10^4^ cells/well and treated with 15 and 30 µM **OI**, respectively, for 24 h. Control cells were added with DMSO and processed at the same time. Then, cells were washed with PBS and stained in the dark with CM-H_2_DCFDA (diluted in PBS/CM to a final concentration of 1 μM) for 20 min at 37 °C. Next steps were also performed in the dark, in order to avoid probe oxidation. Cells were washed three times with PBS, harvested, re-suspended in PBS/CM, and fluorescence was measured by using Fortessa (BD Bioscience, San Jose, CA, USA). ROS levels were estimated by using mean fluorescent intensity of the viable cell population. Results were analyzed by the use of FlowJo software (Tree Star Inc., Ashland, OR, USA).

### 2.9. Tunel Assay

Apoptosis was tested by enzymatic labeling of DNA strand breaks using terminal deoxynucleotidyl transferase-mediated deoxyuridine triphosphate nick end-labeling (TUNEL) assay, following the guidelines of the manufacturer (TUNEL assay kit, Promega, Madison, WI, USA). Briefly, MCF-7 and HeLa cells were added with DMSO or treated with 15 and 30 µM **OI**, respectively, for 72 h. MCF-10A were added to DMSO or treated using 30 µM **OI**, for 72 h. Next, nuclear staining was achieved using 0.2 mg/mL 4′,6- diamidino-2-phenylindole (DAPI, Sigma-Aldrich, Milan, Italy) [40] and samples were examined using a fluorescent microscope (Olympus BX4 equipped with CSV1.14 software employing a CAMXC-30 for image acquisition). Images were representative of three independent experiments.

### 2.10. Statistical Analysis

Data represented means ± standard deviation (SD) considering at least three independent experiments, with ≥ three technical replicates per experiment, except when otherwise stated. Statistical significance was evaluated using analysis of variance (ANOVA) test by GraphPad Prism 7 (GraphPad Inc., San Diego, CA, USA). Dunnett’s test was employed as a post hoc test for pairwise comparisons. All statistical tests were two-sided, and *p* ≤ 0.05 was considered significant.

## 3. Results

### 3.1. Antiproliferative Activity

**Figure 1:**
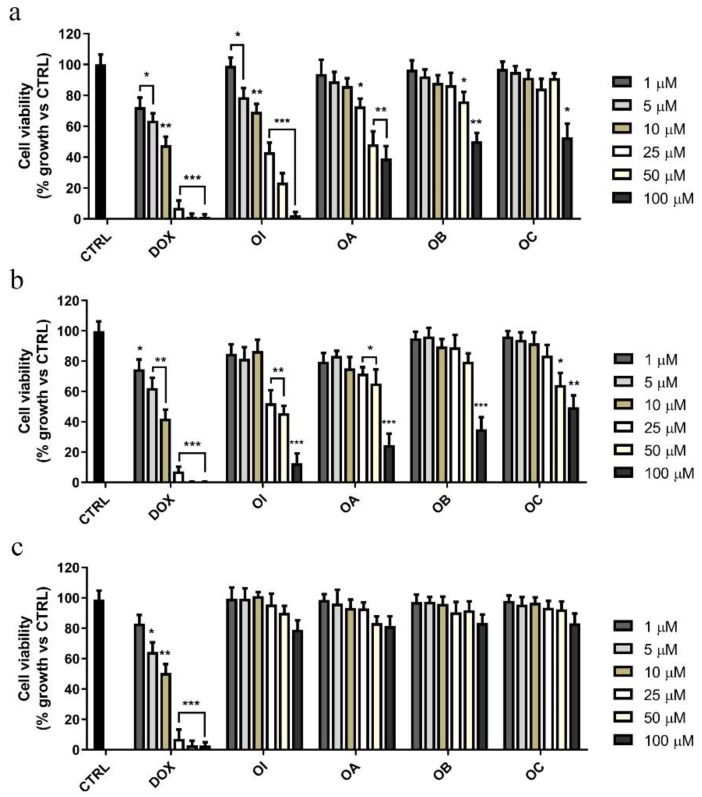
Effects of the compounds OA-OI on MCF-7, HeLa, and MCF-10A cell growth. (**a**) Breast cancer cells (MCF-7), (**b**) human uterine cervix adenocarcinoma cells (HeLa), and (**c**) non-tumorigenic breast epithelial cells (MCF-10A) were treated for 72 h with vehicle alone (control cells, CTRL) or increasing doses (1 to 100 μM) of each compounds or doxorubicin (DOX), as indicated. Cell viability was assessed by MTT assay and was expressed as percentage of growth vs. CTRL. Values represent means ± SD of three different experiments, each performed with triplicate samples. * *p* value < 0.05; ** *p* value < 0.01; *** *p* value < 0.001.

The effects on cancer cell proliferation of the aforementioned compounds were evaluated in two human tumor cell lines, MCF-7 and HeLa, by using the (3-(4,5- dimethylthiazol-2-yl)-2,5-diphenyl tetrazolium bromide) MTT assay. For this purpose, both cell lines were treated with increasing doses (1, 10, 50, 75, and 100 µM) of different oxazolidin-2-one derivatives (**OA–OI**) for 72 h (data not shown). Cells were also exposed to doxorubicin (DOX), in order to compare antiproliferative effects of such derivatives to those of this extensively employed anti-cancer drug. Among all the tested compounds, we found that only 2-oxazolidinones **OA**, **OB**, **OC**, and **OI** displayed an interesting anti-proliferative activity (Figure 1a,b).

In particular, **OI** treatment elicited the highest effect by reducing cell viability in a dose-dependent manner (Figure 1a,b) both in MCF-7 and HeLa cells, with half-maximal inhibitory concentration (IC_50_) values equal to 17.66 and 31.10 µM, respectively (Table 1).

On the other hand, the compounds **OA**, **OB**, and **OC** revealed noticeable inhibitory activity either on MCF-7 or on HeLa cell growth only when they were used at high concentrations (ranging between 50 and 100 µM). It should be underlined that, although **OI** was less active than DOX, as highlighted by its higher IC_50_ values (Table 1), **OI** treatment up to 72 h induced no significant anti-proliferative effect on non-tumorigenic MCF-10A breast epithelial cells (Figure 1c, Table 1). These findings highlighted that **OI** had a more specific inhibitory effect on cancer cells with respect to that exerted on non-tumorigenic cells.

### 3.2. Oxazolidinone OI Induces Cell Cycle Arrest

In order to understand whether **OI** antiproliferative effect could be a consequence of cell cycle perturbations, we assessed flow cytometric cell cycle analysis and estimated the deployment of cells within the three main phases of the cell cycle, by propidium iodide (PI) staining. Both MCF-7 and HeLa cell lines were treated for 24 h with the vehicle alone (DMSO) or with the compound **OI** used at 15 and 30 µM, respectively.

**Figure 2:**
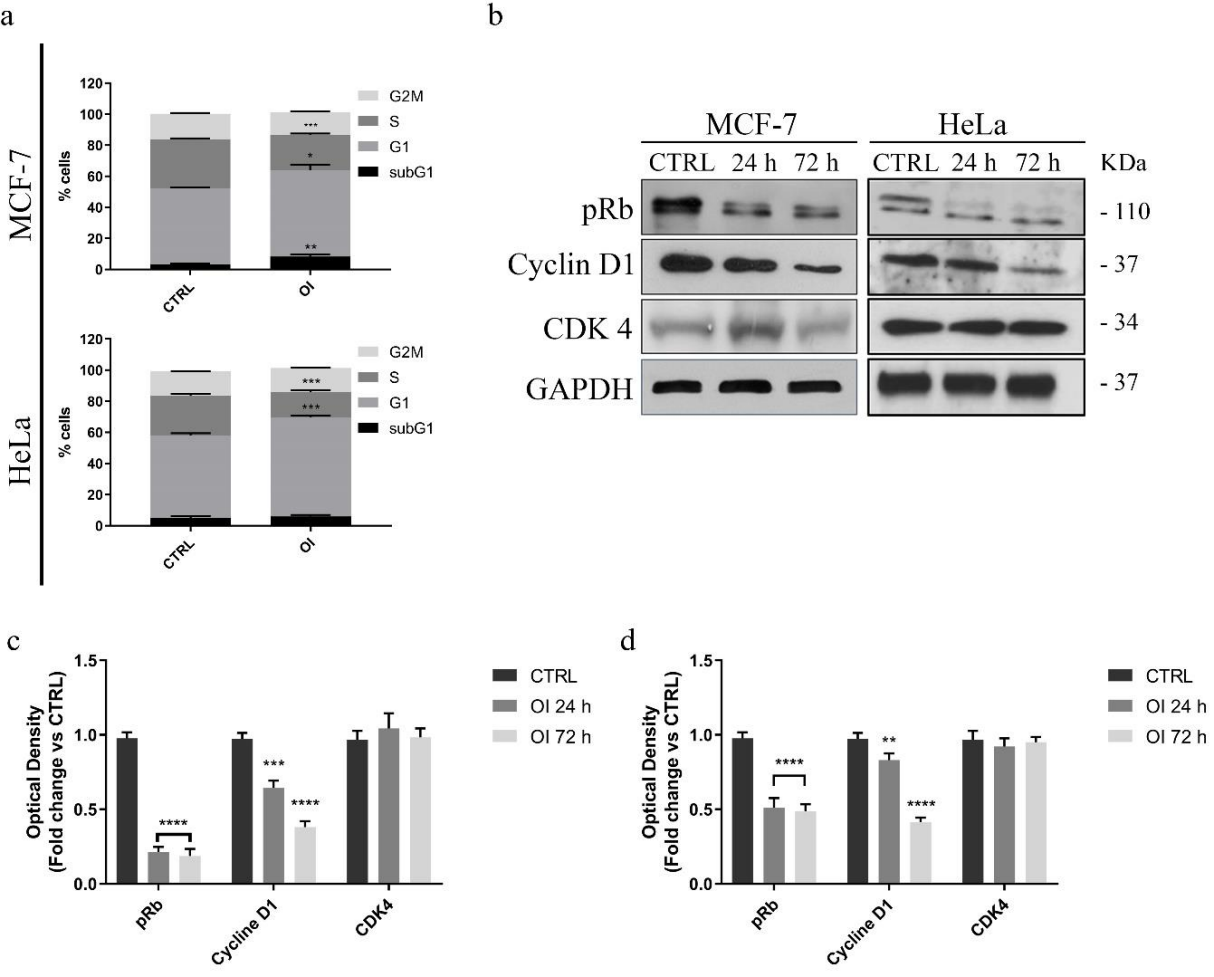
Effects of the compound OI on the cell cycle progression in cancer cells. (**a**) Quantitative analysis of the percentage of cells arrested in different phases of the cell cycle was indicated. Immunoblot and densitometric analyses of several controlling proteins of the G1/S phase transition in MCF-7 (**b**) and HeLa (**c**,**d**) treated cells. GAPDH was used as a loading control. Histograms represent means ± SD of three different experiments in which band intensities were assessed as optical density arbitrary units (OD), and expressed as fold change vs. control samples (CTRL). It is noteworthy that **OI** treatment impairs the G1/S transition of both cancer cell lines. * *p* value < 0.05; ** *p* value < 0.01; *** *p* value < 0.001; **** *p* value < 0.0001.

Our results revealed that this treatment arrested cells in the G0/G1-phase of the cell cycle (Figure 2a), in agreement with the **OI** induced antiproliferative effect shown in Figure 1.

In particular, after 24 h, we observed a significant increase of the percentage of MCF-7 cells in the G0/G1 phase (15%), concomitant with a reduction of the fraction of cells in the S-phase (30%). Similar results were observed in HeLa cells (Figure 2a) since **OI** treatment induced a considerable decrease of cells in the S phase, together with a remarkable increase in the G1 phase. Conversely, we did not evidence any noticeable effect on the cell cycle in MCF-10A non-tumorigenic breast epithelial cells (Figure S1a), indicating that **OI** selectively inhibits cancer cell proliferation by induction of G0/G1 cell cycle arrest.

In order to confirm our findings, we investigated possible changes in the expression levels of proteins involved in the cell cycle regulation, including cyclin D1, CDK4, and phosphorylated retinoblastoma tumor suppressor (pRb) protein. MCF-7 cells were treated with **OI**, used at 15 µM for 24 and 72 h, and whole cell lysates were subjected to immunoblotting analysis. Consistently with the observed G1/S transition arrest of the cell cycle, this treatment significantly reduced cyclin D1 and phosphorylated Rb protein content in a time dependent manner, whereas CDK4 protein expression levels remained unaffected (Figure 2b,c). Similar results were also observed on 30 µM **OI**-treated HeLa cells, under the same experimental conditions (Figure 2b,d). These data suggest that **OI** treatment arrest cells in the G1-S phase of the cell cycle by inhibiting cyclin D1 expression and Rb phosphorylation. In addition, both these events were not dependent on cell type.

### 3.3. OI Triggers Apoptotic Cell Death in MCF-7 and HeLa cells

**Figure 3:**
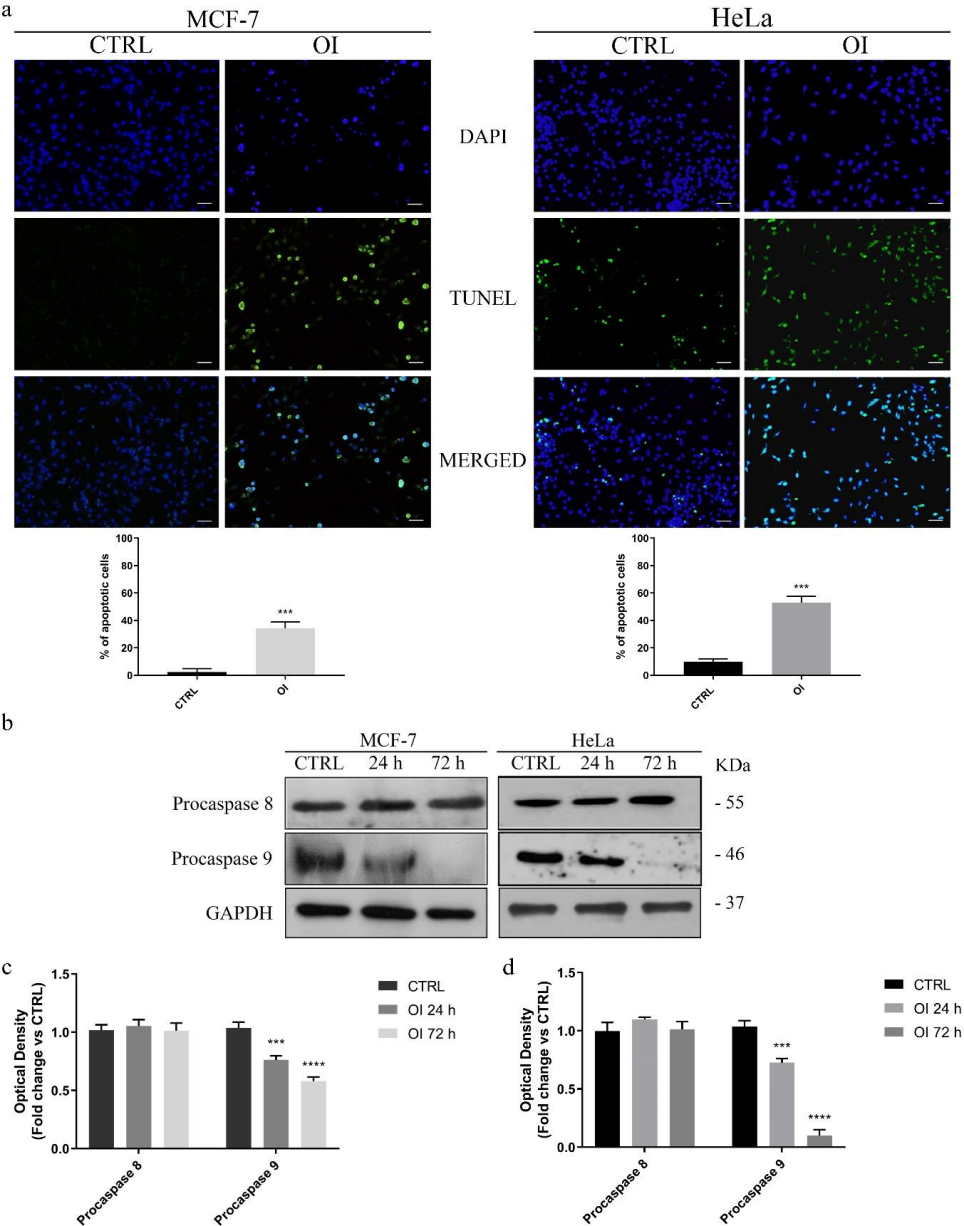
OI treatment triggers cell death by apoptosis. (**a**) TdT-mediated dUTP nick-end-labeling (TUNEL) assay in MCF-7 and HeLa cells treated for 72 h with DMSO (CTRL) or 15 and 30 µM **OI**, respectively. DAPI was used to visualize the cell nucleus. Scale Bars 50 µm. Histograms represent means ± SD of apoptotic cells from three independent experiments. (**b**) Immunoblot analysis of pro-caspases 8 and 9 after 24 and 72 h of **OI** treatment (see Materials and Methods, par. 2.6). GAPDH was used as a loading control. Densitometric quantification of pro-caspase expression levels in MCF-7 (**c**) and HeLa (**d**) treated cells vs. control cells. Remarkably, **OI** treatment induces apoptosis by activating the intrinsic caspase-dependent apoptotic pathways. Values are means ± SD of three different experiments. *** *p* value < 0.001; **** *p* value < 0.0001.

Since several reports evidenced that oxazolidinones were able to induce apoptosis [18,23,24], we next tested whether also **OI** exposure could induce cell death by triggering apoptosis, in both our tested cell lines. The impact of this oxazolidin-2-one derivative on apoptosis was assessed by using two different approaches. Firstly, TUNEL assay was performed in order to test **OI** ability to induce DNA fragmentation, which is a late event of apoptosis. Hence, MCF-7 and HeLa cells were incubated for 72 h with 15 or 30 µM **OI**, respectively, prior to analysis. MCF-10A cells were incubated for 72 h with 30 µM **OI**, the highest concentration used for cancer cells, prior to analysis. Our results evidenced an increased number of TUNEL-positive cells (Figure 3a,b). In control cells (cells treated with DMSO; CTRL), apoptotic population was 2.3% in MCF-7 and 10% in HeLa cells; after the treatment, the apoptotic rate reached 34.3% and 53.2% in MCF-7 and HeLa cells, respectively (Figure 3a). Conversely, we did not evidence any noticeable effect on MCF-10A, non-tumorigenic breast epithelial cells (Figure S1b).

Secondly, apoptosis onset was investigated by testing the activation of caspases -8 and -9, which are two well-known biochemical markers of programmed cell death. For this purpose, MCF-7 and HeLa cells were treated with 15 or 30 µM **OI**, respectively, both for 24 and 72 h, and expression levels of procaspases -8 and -9 were assessed by Western blot analysis (Figure 3b). Our results showed that **OI** treatment time-dependently activated proteolytic cleavage of inactive pro-caspase-9 into its active form, whereas expression level of activated caspase-8 was unaffected (Figure 3b–d). Since the extrinsic apoptotic pathway includes caspase-8 activation, while caspase-9 is involved in the intrinsic mitochondria pathway [30], our findings prompted us to hypothesize that **OI** compound could activate the mitochondrial caspase-dependent apoptotic pathway, in both MCF-7 and HeLa cells.

### 3.4. OI Reduces Mitochondrial Membrane Potential with a Significant Increase in ROS Levels

**Figure 4:**
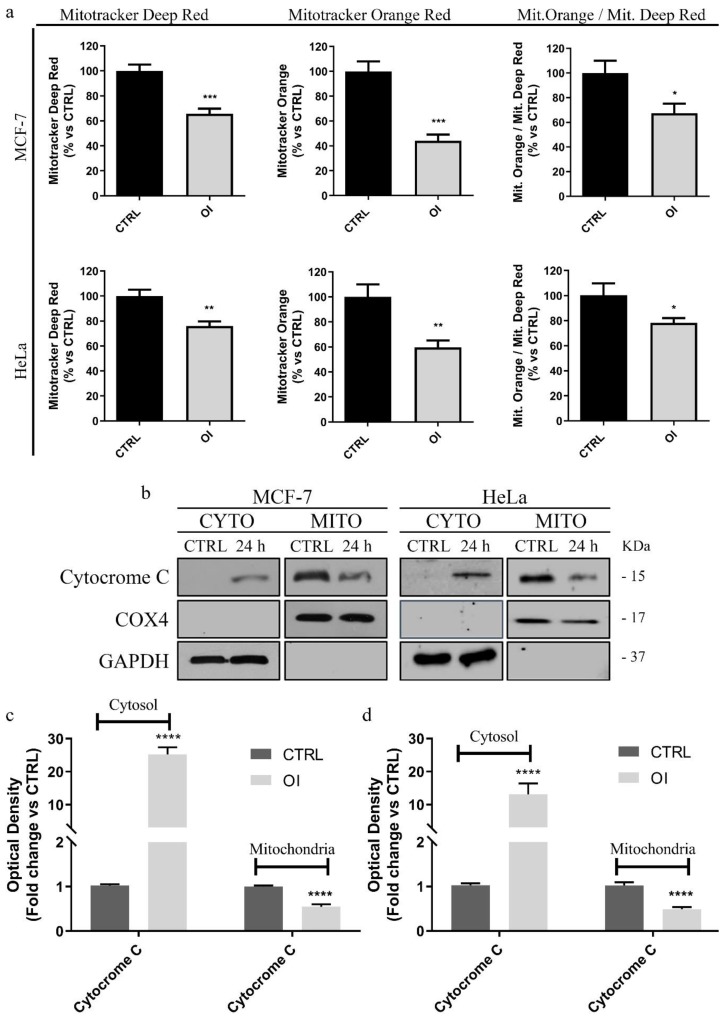
OI treatment induces mitochondrial damage in cancer cells. (**a**) Both MCF-7 and HeLa cells were treated for 24 h either with DMSO (CTLR) or 15 and 30 µM **OI**, respectively, then they were stained with different metabolic probes and analyzed by FACS. Note that the treatment decreases mitochondrial mass (MitoTracker Deep-Red) and mitochondrial membrane potential (MitoTracker Orange). Assessment of mitochondrial membrane potential vs. mitochondrial mass displayed that **OI** decreases mitochondrial membrane potential per mitochondria. Immunoblot and densitometric analysis of cytochrome c in treated MCF-7 (**b**,**c**) and HeLa (**b**,**d**) cells. GAPDH was used as a loading control of cytosolic fractions, whereas COX4 of the mitochondrial one. A significant release of cytochrome c into the cytosol is revealed in both tested cancer cell lines. Histograms represent means ± SD of three independent experiments in which band intensities were assessed as optical density arbitrary units (OD) and expressed as fold change vs. control samples (CTRL). **** *p* value < 0.0001.

In order to validate that **OI** treatment was able to trigger apoptosis cell death by activating the intrinsic mitochondrial pathway, we tested if this compound could induce mitochondrial damage. To this end, we investigated **OI** ability to induce a loss of mitochondrial membrane potential leading to alterations in mitochondrial permeability, which resulted in the release of cytochrome c from the mitochondria (where it is habitually located) into the cytosol. Remarkably, this latter event represents a hallmark of the apoptotic process [41]. Hence, both MCF-7 and HeLa cells were treated for 24 h with 15 or 30 µM **OI**, respectively, stained with specific metabolic probes, and analyzed by fluorescence-activated cell sorting (FACS). Our results showed that **OI** treatment was able to decrease mitochondrial membrane potential as evaluated using MitoTracker Orange (Figure 4a), as well as mitochondrial mass, as demonstrated by MitoTracker Deep-Red. (Figure 4a). The ratio of mitochondrial membrane potential (MitoTracker Orange) vs. mitochondrial mass (MitoTracker Deep-Red) was also calculated, showing that **OI** treatment significantly decreased mitochondrial membrane potential per mitochondria (Figure 4a) in MCF-7 as well as in HeLa cells (Figure 4a). Additionally, Western blotting analysis performed on the lysates of cells treated under the same experimental conditions confirmed cytochrome c release. Indeed, significant increases in cytochrome c levels in the cytosolic fraction, together with concomitant decreases in its mitochondrial levels, were found in both MCF-7 (Figure 4b,c) and HeLa (Figure 4b,d) **OI**-treated cells, when compared to the control cells.

**Figure 5:**
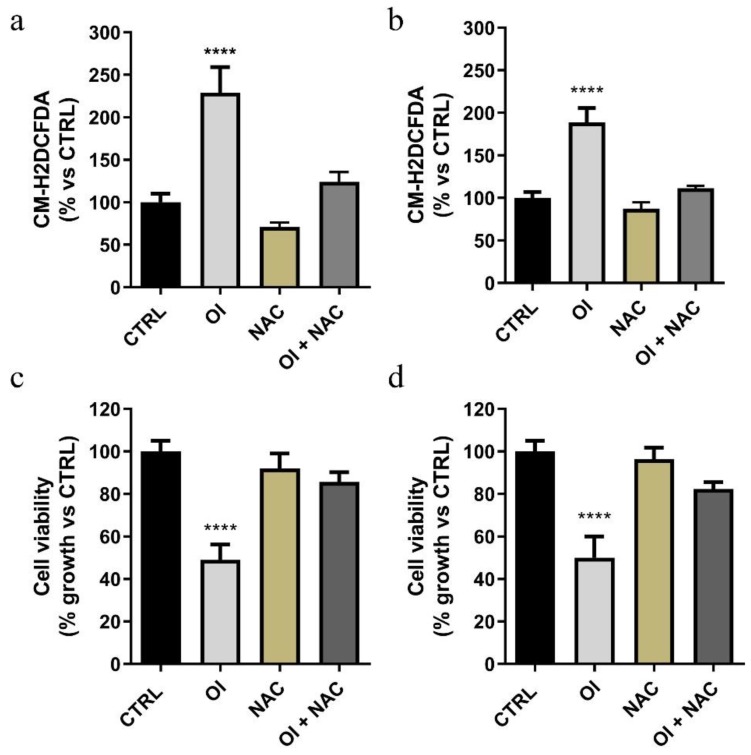
The compound OI causes a significant increase in ROS levels. Both MCF-7 (**a**) and HeLa (**b**) cells were treated for 24 h with 15 and 30 µM **OI**, respectively, alone and/or in the presence of N-acetyl-l-cysteine (NAC); ROS levels were evaluated by FACS analysis using the CM-H2DCFDA probe. Cell proliferation assay (MTT assay) was performed in both treated MCF-7 (**c**) and HeLa (**d**) cells, in the presence or absence of 3 mM NAC. Note that after 72 h, in the presence of NAC, cells displayed recovery in growth. Values represent means ± SD of three different experiments, each performed with triplicate samples. * *p* value < 0.05; **** *p* value < 0.0001.

It is known that reactive oxygen species (ROS) are referred as being mediators of apoptosis, particularly due to their great reactivity towards various macromolecules involved in the electrochemical mitochondrial membrane balance [42,43]. On this basis, we investigated whether ROS production could underlie **OI**-induced apoptosis (Figure 5a,b).

Both MCF-7 and HeLa cells were treated for 24 h with 15 and 30 µM **OI**, respectively, alone or in the presence of a ROS quencher such as *N*-acetyl-l-cysteine (NAC). Then, ROS levels were evaluated by FACS analysis using the CM-H2DCFDA probe. We found that this treatment significantly stimulated ROS generation in both the tested cell lines, when compared to the control cells (Figure 5a,b). Furthermore, the observed increases were no longer evident in the presence of NAC (Figure 5a,b). Finally, we proved that the anti-proliferative activity evidenced after **OI** exposure was strictly dependent on ROS generation, as highlighted by the increased cell viability found in both cell types in the presence of NAC (Figure 5c,d). Indeed, MCF-7 and HeLa cells exhibited resistance to **OI** when co-incubated for 72 h with 3 mM NAC, showing 40% (Figure 5c) and 35% (Figure 5d) recovery in growth, respectively. These last data supported our hypothesis, according to which ROS production could underlay mitochondrial damage, cytotoxicity, and cell death induced by the compound **OI**.

## 4. Discussion

Despite the fact that glycolysis has long been believed to be a key metabolic pathway for anabolic growth and energy production in cancer cells, it is currently evident that mitochondria play a critical role in oncogenesis by affecting all its steps [44]. Mitochondria are involved in bioenergetic metabolism, as well as providing specific metabolites that support oncogenesis [45]. Several mitochondrial metabolic pathways may adjust in order to modulate anabolic or bioenergetic functions, thus conferring a great metabolic plasticity to tumor cells [46,47]. These organelles supply pivotal components for redox control and participate in transcriptional regulation; moreover, mitochondrial membranes permeabilization represents a crucial step in the programmed cell death [48,49,50,51]. Remarkably, mitochondrial function proved to be essential for an effective formation and propagation of CSCs [52], which are a sub-population of cancer cells responsible for initiation, development, and cancer recurrence; furthermore, they are usually resistant to conventional radio- and chemotherapy [53,54,55,56,57]. In this light, recently, different researches have been focused on developing several molecules endowed with antineoplastic properties and able to target mitochondria, in order to find new future anticancer candidates [58,59,60].

Here, we proved the ability of the synthetic 5-(carbamoylmethylene)-oxazolidin-2-one derivatives (**OA–OI**) to induce antiproliferative activity in two different cancer cell lines, MCF-7 and HeLa cells, without affecting the proliferation of the non-tumorigenic MCF-10A cells. Among the tested compounds, only **OA**, **OB**, **OC**, and **OI** displayed an interesting anti-proliferative activity. Remarkably, **OI** emerged as the most promising anticancer agent, since it showed the lowest IC_50_ value in both the tested cancer cell lines. **OI** treatment was also associated with apoptosis induction, which was triggered by increased ROS levels and mitochondrial damage.

Cell cycle alterations leading to abnormal cell proliferation represent a common cancer feature [61]. Therefore, a promising anticancer agent should be able to target regulatory elements of the cell cycle in order to block it, so preventing tumor proliferation. Cyclins and CDKs exert a critical function in regulating the cell cycle progression [62]. In detail, Cyclins D plays a key role in the transition from the G1 to the S phase, and the G1 progression requires cyclin D1-CDK4/6 complex formation [63]. Additionally, activated cyclin/CDK complexes phosphorylate the retinoblastoma (Rb) tumor suppressor protein, which promotes the cell cycle transition from the G1 to the S phase [64]. By using cytofluorimetric and immuno-detection analyses, we proved that **OI** treatment was associated with an impaired cell cycle progression. In detail, we observed a cell cycle arrest in the G1 phase together with a reduced percentage of cells in the S phase, in a time dependent manner. At the same time, we found concomitant decreased expression levels of both cyclin D1 and phosphorylated Rb protein.

Scientific literature data reported that different oxazolidin-2-one derivatives could inhibit cell proliferation and promote apoptosis in a variety of cancer cell lines [18,23,24]. On this basis, we explored **OI** ability to induce apoptosis in MCF-7 and HeLa cells. Our findings proved that this compound was able to trigger apoptosis, as highlighted by a significant increase in the percentage of TUNEL-positive cells and by a concomitant decrease in procaspase-9 levels observed in both cell lines, when compared to the control cells. Conversely, procaspase-8 expression levels were unaffected.

Thus, our findings support the hypothesis that **OI** treatment is able to inhibit cell cycle progression and to trigger the intrinsic apoptotic mechanism through the activation of procaspase-9 in initiator caspase-9, which is involved only in this apoptotic pathway [65]. Accordingly, we found no decrease in procaspase-8 protein levels, proving a failed activation of the initiator caspase-8, highlighting that the extrinsic apoptotic pathways is not implicated in cell death [65]. Moreover, in good agreement with our hypothesis, we detected, by Western blot analysis, a noticeable increase in cytochrome c release from mitochondria into the cytosol, which is a key event in the caspase-dependent mitochondrial pathway. Indeed, within the cytosol, cytochrome c binds to apoptotic protease-activating factor-1 (Apaf-1) producing the apoptosome complex. The latter recruits and activates the initiator procaspase-9, which in turn induces cleavage/activation of the downstream effector caspases-3 and -7 [43]. Mitochondria-to-cytosol translocation of cytochrome c is caused by a mitochondrial damage due to a loss of mitochondrial membrane potential, followed by mitochondrial membrane permeabilization [66]. In this context, our results clearly demonstrated that **OI** treatment significantly reduced mitochondrial membrane potential, confirming its ability to produce mitochondrial damage, as well as to trigger cell death by activating the intrinsic apoptotic pathway.

Mitochondrial dysfunction was also confirmed by a decrease in the mitochondrial mass detected after **OI** treatment. In this regard, it is known that mitochondrial mass affects mitochondrial function, and it depends on turnover and biogenesis, two metabolic processes acting as tumorigenesis regulators [67]. Reactive oxygen species (ROS) are generated by several metabolic pathways, and their role is determined by their concentration. At physiologically low levels, they act as “redox messengers” in intracellular regulation, whereas their overproduction can induce cell death [43]. Moreover, ROS are considered mediators of apoptosis, mainly because of their involvement in mitochondrial outer membrane permeabilization and in the translocation of pro-apoptotic mitochondrial proteins into the cytosol [42,43]. As a consequence, we investigated whether **OI** could induce an increase in ROS levels, which in turn could trigger apoptotic cell death. Our outcomes demonstrated that **OI** treatment increased overall ROS production. Noteworthily, we found increased cell viability when this treatment was performed in the presence of NAC, suggesting that high ROS levels, together with mitochondrial damage and cell death, could be responsible for **OI** anti-proliferative activity.

Taken together, our findings highlight that the tested oxazolidin-2-one derivative induce cell death through the activation of the intrinsic mitochondrial pathway in MCF-7 and HeLa cells, without exerting any effects on non-tumorigenic MCF-10A breast epithelial cells. In conclusion, the compound **OI** might represent a promising drug candidate for future therapies in cancer treatment, as well as the fact that it can be considered a very promising starting point for future chemical syntheses in order to find new molecules endowed with potential anticancer properties.

## Figures and Tables

**Table 1:** Cytotoxic activity of 5-(Carbamoylmethylene)-oxazolidin-2-ones in comparison to that of doxorubicin ^a^.

Compound	HeLa		MCF-7		MCF-10A	
	IC_50_	95% Confidence Interval	IC_50_	95% Confidence Interval	IC_50_	95% Confidence Interval
DOX	3.61	3.02 to 4.32	4.04	3.25 to 5.03	31.75	25.16 to 40.08
OA	52.53	42.81 to 64.45	56.90	50.43 to 69.18	414.3	336.3 to 510.5
OB	98.21	81.46 to 118,0	121.8	107.0 to 139.7	469.6	378.9 to 582.1
OC	99.47	88.42 to 111.9	163.3	135.2 to197.2	494.5	406.2 to 602.2
OD	154.5	136.4 to 175.1	338.4	284.8 to 402.0	506.4	410.1 to 625.4
OE	129.3	114.0 to 146.0	303.7	258.3 to 357.0	978.2	718.3 to 1332
OF	159.4	138.5 to 183.6	409.3	348.8 to 480.3	718.4	506.6 to 1019
OG	134.4	113.5 to 159.2	344.9	290.4 409.6	677.1	530.3 to 864.5
OH	54.21	42.67 to 68.88	92.91	78.99 to 109.3	318.8	263.1 to 386.3
OI	31.10	26.29 to36.80	17.66	15.67 to 19.91	351.3	288.4 to 427.8

^a^ Data are presented as IC_50_ values (µM) and 95% confidence intervals obtained by nonlinear regression analysis of three independent experiments.

## Supplementary Materials

Supplementary materials can be found at https://www.mdpi.com/2227-9059/8/2/35/s1.
