# Supplementary material for: 5-(Carbamoylmethylene)-oxazolidin-2-ones as a Promising Class of Heterocycles Inducing Apoptosis Triggered by Increased ROS Levels and Mitochondrial Dysfunction in Breast and Cervical Cancer
Source: Biomedicines. 2020 Feb 18;8(2):35. doi: 10.3390/biomedicines8020035 (PMC7168333; doi:10.3390/biomedicines8020035)
Supplement: Supplementary file 1 [file biomedicines-08-00035-s001.pdf]

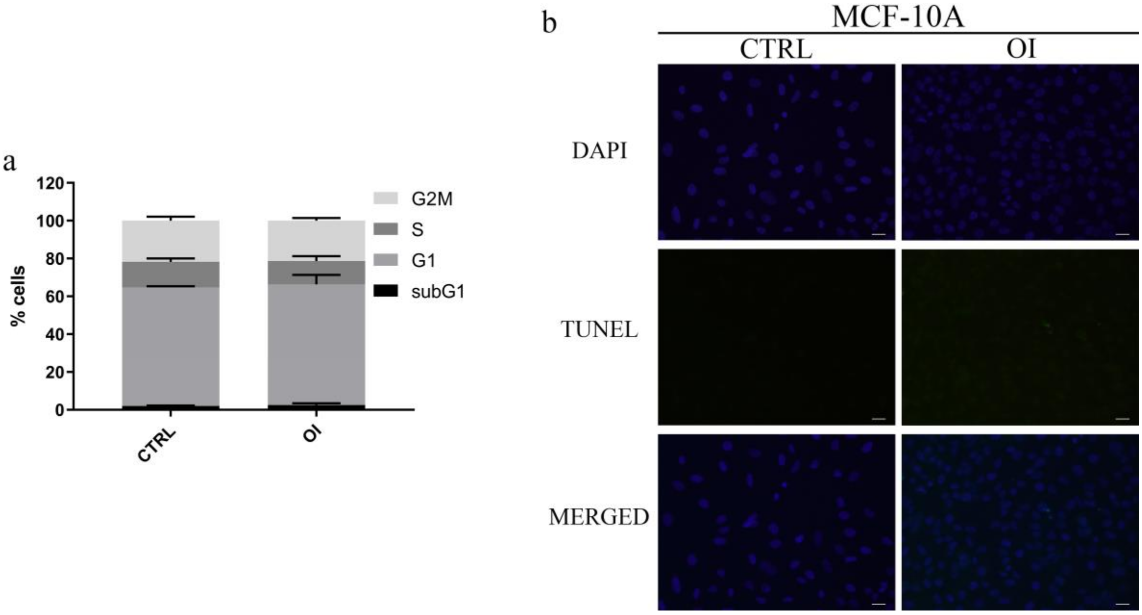

Figure S1. OI does not determine cell cycle arrest and cell death in MCF-10A cells. (**a**) Quantitative analysis of the percentage of cells in different phases of the cell cycle was indicated. Histograms represent means ± SD of three different experiments. (**b**) TdT-mediated dUTP nick-end-labeling (TUNEL) assay in MCF-10A cells treated for 72 h with DMSO (CTRL) or 30 μM OI. DAPI was used to visualize the cell nucleus. Scale Bars 25 μm.
